# Neuropathological and Reelin Deficiencies in the Hippocampal Formation of Rats Exposed to MAM; Differences and Similarities with Schizophrenia

**DOI:** 10.1371/journal.pone.0010291

**Published:** 2010-04-22

**Authors:** Julien Matricon, Alfredo Bellon, Helge Frieling, Oussama Kebir, Gwenaëlle Le Pen, Frédéric Beuvon, Catherine Daumas-Duport, Thérèse M. Jay, Marie-Odile Krebs

**Affiliations:** 1 INSERM U894, Laboratoire de Physiopathologie des Maladies Psychiatriques, Centre de Psychiatrie et Neurosciences, Paris, France; 2 Université Paris Descartes, Faculté de Médecine Paris Descartes, Hôpital Sainte-Anne, Paris, France; 3 Department of Psychiatry, Socialpsychiatry and Psychotherapy, Hannover Medical School, Hannover, Germany; 4 Neuropathology unit, Université Paris Descartes, Faculté de Médecine Paris Descartes, Hôpital Sainte-Anne, Paris, France; 5 INSERM U894, Laboratoire de Plasticité gliale et tumeurs cérébrales, Centre de Psychiatrie et Neurosciences, Paris, France; Chiba University Center for Forensic Mental Health, Japan

## Abstract

**Background:**

Adult rats exposed to methylazoxymethanol (MAM) at embryonic day 17 (E17) consistently display behavioral characteristics similar to that observed in patients with schizophrenia and replicate neuropathological findings from the prefrontal cortex of psychotic individuals. However, a systematic neuropathological analysis of the hippocampal formation and the thalamus in these rats is lacking. It is also unclear if reelin, a protein consistently associated with schizophrenia and potentially involved in the mechanism of action of MAM, participates in the neuropathological effects of this compound. Therefore, a thorough assessment including cytoarchitectural and neuromorphometric measurements of eleven brain regions was conducted. Numbers of reelin positive cells and reelin expression and methylation levels were also studied.

**Principal Findings:**

Compared to untreated rats, MAM-exposed animals showed a reduction in the volume of entorhinal cortex, hippocampus and mediodorsal thalamus associated with decreased neuronal soma. The entorhinal cortex also showed laminar disorganization and neuronal clusters. Reelin methylation in the hippocampus was decreased whereas reelin positive neurons and reelin expression were unchanged.

**Conclusions:**

Our results indicate that E17-MAM exposure reproduces findings from the hippocampal formation and the mediodorsal thalamus of patients with schizophrenia while providing little support for reelin's involvement. Moreover, these results strongly suggest MAM-treated animals have a diminished neuropil, which likely arises from abnormal neurite formation; this supports a recently proposed pathophysiological hypothesis for schizophrenia.

## Introduction

Accumulating evidence suggests that schizophrenia originates from abnormal central nervous system (CNS) development [1], [2]. Data supporting this premise come in particular from cytoarchitectural studies of patients in whom heterotopias, laminar disorganization and neuronal clusters in the hippocampal formation have been reported [3]–[7]. Although these findings have not been always replicated [8]–[10], other anatomical and neuromorphometric changes in affected individuals are more consistent. Among such deficiencies are a smaller hippocampus and entorhinal cortex, both of which are associated with reduced neuronal body sizes [11]–[19]. Neuropathological and electrophysiological studies support GABAergic interneurons as a key actor in the pathophysiology of schizophrenia [20], [21]. In the prefrontal cortex (PFC) and in the hippocampus these neurons express reelin [22] and previous studies have consistently found reelin's expression to be decreased in the PFC, hippocampus, caudate nuclei and cerebellum of patients with this psychotic illness [23], [24]. Other common findings include a decreased number of reelin-containing cells in the PFC, cerebellum and hippocampus [23]–[25] as well as hypermethylation of the reelin promoter [26]–[28], although the most recent study did not replicate this latter finding [29].

In accordance with the neurodevelopmental hypothesis of schizophrenia [1], [2], exposure to Methylazoxymethanol acetate (MAM) during rats' embryonic life has been used to model this psychotic illness. Several embryonic days have been tested, ranging from embryonic days (ED) 9 to 12 [30]–[32] and most recently embryonic day 17 [33]–[35]. MAM, an anti-mitotic alkylating agent [36], is injected into pregnant rats to disrupt the pups' CNS development. This compound induces programmed cell death in neuroepithelial proliferating cells [37] without causing any teratogenic effects in any other organs [38]. Embryonic day 17 (E17) was selected because it is when neurogenesis peaks in the hippocampal formation [39] and when projections from the hippocampus to the entorhinal cortex are established in rats [40]. Since the hippocampal formation is considered central for the pathology of schizophrenia [41], disrupting this brain region's development seems crucial to model such an illness. MAM exposure earlier in embryonic development also affects the hippocampal formation [30]–[32] but it is adult rats exposed to MAM at E17 which display behaviors that resemble some characteristics observed in schizophrenia such as social withdrawal [42], [43], hypersensitivity to amphetamines and NMDA-antagonists [42]–[44], sensory gating anomalies [43], [45] and cognitive impairments [35], [42], [43], [46]. Less information, however, is currently available regarding possible neuropathological anomalies present in these animals.

Three independent groups have reported cortical and hippocampal size reductions in MAM E17 rats [35], [42], [47]. More recently, the MAM E17 model was found to reproduce whole neuronal density changes from the PFC and parvalbumin-positive-cell density deficits from the hippocampus and the PFC of patients with schizophrenia [45], [48]. Along with these findings, a smaller mediodorsal thalamic nucleus and a smaller hippocampus were described [45] but further analysis of these brain regions involving neuronal density, size, morphology and localization were not pursued. Consequently, the first goal of our work was to determine the neuropathological substrate of the hippocampal formation and thalamus size reductions in the MAM model and to establish if such a neuropathological substrate correlates with deficiencies observed in patients with schizophrenia. In order to achieve this goal, we conducted a thorough examination of the hippocampus (HIP), dentate gyrus (DG), entorhinal (ERC) and perirhinal cortices (PER), thalamus (THAL), mediodorsal thalamus (MDT), somatosensory barrel field 1 (S1BF) and associative parietal cortex (PtA), piriform cortex (PIR), amygdala (AMG), ventral tegmental area (VTA) and substantia nigra (SN). Factors identified included differences in whole structural sizes as well as changes in neuronal density, size, shape, organization and localization. The second goal of this work was to determine if reelin's expression and methylation levels as well as the number of reelin-positive cells are affected in the hippocampal formation of MAM treated animals. Reelin was selected for investigation due to its consistent association with schizophrenia [23]–[27] and because it can affect neuronal morphology, size and migration [49], [50], [51]. Moreover, MAM's mechanism of action could be related to its alkylating effects over the reelin gene.

## Methods

### Animals

Thirty pregnant Sprague-Dawley dams (Charles River, France) were obtained at embryonic day 10 and housed individually. On E17, the dams were treated with methylazoxymethanol 25mg/kg (Midwest Research Institute, Kansas City, MO, USA) or saline solution intraperitoneally (i.p., 1ml/kg). MAM and sham litters were grouped respectively. Rats born from saline-injected pregnant rats served as controls. Pups were weaned twenty-one days after birth and placed into cages suitable for two or three animals. Rats were kept on a 12-hour light/dark cycle and received water and food *ad libitum* before being sacrificed as adults for immunohistochemistry. A second batch of eleven pregnant Sprague-Dawley animals treated identically was later used to study reelin methylation and expression levels in the hippocampus. All experiments had the local Ethics Committee agreement number P2.TJ.104.09 (Comité regional d'éthique pour l'expérimentation animale, Ile-de-France – René Descartes) and were performed following the French Agriculture and Forestry Ministry regulations (decree 874848, license A91429) which are in accordance with the European Union guidelines concerning the care and use of laboratory animals.

### Brain removal and storage

At six months of age, 26 rats (13 males and 13 females) from the first batch and 14 female rats from the second batch were sacrificed via CO_2_ inhalation and their brains were removed from their skulls. For the first batch of animals, brains were fixed in formol-Zn at 4°C for 24 hours. Before the embedding cycle, which consists of increasing alcohol baths for tissue dehydration, toluene was used as a solvent and finally paraffin was applied. For the second batch of 11 animals the hippocampus was dissected and the procedures described in the reelin methylation and expression sections were followed.

### Stainings and immunohistochemistry

Paraffin-embedded brains from 14 control and 12 MAM-exposed animals were cut in 5 µm slices with a microtome Microm HM340E and fixed on Superfrost Plus slides (Menzel-Gläser) in parallel series. From the 14 control brains and the 12 MAM-exposed brains, 9 (5 males and 4 females) from each group were included in the analyses. The remaining brains were not suitable for analysis due to technical difficulties. Two levels were chosen involving the hippocampus; Bregma −3.4 to −3.8 mm and Bregma −5.3 to −5.7 mm. 60 slices were made for each level and 3 to 5 sections were studied per level. After an overnight passage in the drying oven, slices were conserved at 4°C. Brain slices' adhesion to glass slides was improved by a two minutes passage in the microwave oven.

Immunolabelling was performed using a protocol based on the avidin-biotin-peroxidase system based on the Vectastain ABC kit (Vector Laboratories). Endogenous peroxidase activity was quenched by incubating the brain slices for 15 minutes in 0.3% hydrogen peroxide. Antigenic sites were revealed by a microwave cycle in a citrate buffer. Slides were then mounted on cover slips and incubated with anti-NeuN (Neuronal Nuclei, 1∶500), anti-GFAP (GlioFibrilar Acid Protein, 1∶200) or anti-reelin (1∶1000) antibodies for an hour at room temperature. Then, sections were incubated in avidin-biotin complex (Vectastain ABC kit, Vector Laboratories) for 30 minutes and 3,3′-diaminobenzidine (Dakocytomation) was used as a chromogen. Slices were washed with distillate water, counterstained with hematoxylin, dehydrated with alcohol and mounted in Merckoglass medium (Merck).

### Morphometric Analysis

*Microscopy*. Our preparations were analyzed on a Nikon eclipse E600 type 120 light microscope at magnifications of 20×. Quantifications at 100×, 200× and 400× and the numerical acquisition of images were performed on a Nomarsky Zeiss Axioplan brightfield microscope and a Nikon Dig Cam DXM1200 connected to a PC unit. *Thickness and area measurements*. Area measures from the HIP, DG, THAL, MDT, AMG, SN, VTA, ERC and the lateral ventricles as well as thickness for the S1BF, PtA, ERC, PER, and PIR cortices were identified using anatomic positions relative to cerebral structures described by The Rat Brain Atlas [52]. *Heterotopias and discontinuities*. Heterotopias, defined as a group of misplaced neurons, were counted in the hippocampus from MAM and control animals. Discontinuities were defined as a disruption in the Cornu Ammonis (CA) layers characterized by the presence of an interruption in the neuronal layer continuity and dispersed neurons on any side of the cellular layer. *Neuronal density assessments*. Neuronal density was calculated as the number of neurons divided by the area (mm^2^) of interest. At Bregma −3.6 to −3.8 mm neuronal density was determined in the S1BF, MDT, PER, ERC, CA1, CA2, CA3, CA4 and DG. Note that CA4 corresponds to the polymorphic layer of the dentate gyrus [53]. This substitution was made to facilitate the comparison with postmortem brains from patients with schizophrenia. *Area, thickness and neuronal number quantification*. Area, thickness and neuronal number measurements were made with the image analysis software Lucia G (Nikon, France). At least 8 rats were used for each group and at least four mean measurements (n = 4–6) for each animal were used to analyze inter-group variations. *Neuronal size measurement*. Sections stained with antibodies against the neuronal marker Neu-N were used for neuronal size measurements. Neuronal size in the ENT, S1BF and MDT was determined with an algorithm from the image analysis software Metamorph (Metamorph, Molecular Devices, France). First, pictures were calibrated according to the objective used in the microscope. Second, we used the Metamorph morphology filter to obtain the background in white (parameter ‘Suppress dark objects’) and the objects in black (parameter ‘Border objects’). This filter permits the software to accurately discriminate objects from background. Then, an ‘inclusive threshold’ was adjusted so as to select the entire body of neurons. The background noise was eliminated by defining the minimum number of pixels necessary to consider them as an object (parameter ‘Pixel area’ arbitrarily defined as 200). In addition, neurons were identified by Metamorph through shape (‘shape factor’ arbitrarily defined as 0.2) and length (‘Length’ defined as >10µm). These settings were validated through the comparison between manual neuronal counts in the original picture, manual neuronal counts in the ‘thresholded’ picture and the result of neuronal counts provided by Metamorph. No difference was seen between these different procedures. Finally, the Metamorph tool ‘Integrated morphometry analysis’ was used to calculate the neuronal soma area selected by the algorithm. Neuronal density in hippocampal layers did not allow the individualization of neurons. Cross-sectional areas of neuronal somas were thus measured manually in CA1, CA2, CA3, CA4 and DG.

### Methylation

Hippocampal Deoxyribonucleic acid (DNA) from the second batch of 11 rats was treated with sodium bisulfite using the EpiTect Kit ® (Epigenomics, Qiagen, BBBB). Three brains from this batch of animals were not suitable for analysis due to technical difficulties. Briefly, 1µg of DNA in a volume of 20µl was mixed with 35µl of DNA Protect Buffer and 85µl of Bisulfite Mix. Bisulfite conversion was achieved using 3 cycles of denaturation (5 minutes at 99°C) and incubation (25, 85, and 175 min respectively at 99°C). Modified DNA was purified by EpiTect ® spin columns and used immediately or stored at −20°C. Bisulfite-modified genomic DNA was amplified with primers specific for the reelin promoter (5′-CGTTTTTTTATTTTGGTTTGGT-3′ (forward), and 5′-TCATATCATACATAACCACTATCCCTA-3′ (reverse). The polymerase chain reaction (PCR) conditions were 95°C for 15 minutes, 5 cycles of 95°C for 30 s, 56°C for 90 s, 72°C for 120 s, 25 cycles of 95°C for 30 s, 56°C for 90 s, 72°C for 90 s, and finally 7 minutes at 72°C. The PCR mixture contained 12.5µl of HotStarTaq Mix (Qiagen), 0.5µl of each primer at a 20µM concentration, and 2µl bisulfite modified DNA. These first-round PCR products were then used as template (1µl) and reamplified by tagged primers (5′-CCACTCACTCACCCACCC+forward primer-3′ and 5′-GGGTGGGAGGTGGGAGGG+reverse primer-3′). The second-round PCR conditions were 95°C for 15 min, 35 cycles of 95°C for 30 s, 55°C for 90 s, 65°C for 120 s, and finally 7 min at 65°C. PCR products were then purified with a Gel Extraction Kit (Qiagen) and subjected to direct cycle sequencing on an ABI3100Avant automated DNA sequencer (Applied Biosystems, Foster City, CA, USA), using the primers from the 2^nd^ round of PCR. Sequence chromatographs were analyzed by the software ESME® (Epigenomics), which performs quality control, normalizes signals, corrects for incomplete bisulfite conversion, and maps positions in the trace file to CpG in the reference sequence.

### Expression

Total Ribonucleic acid (RNA) from hippocampal samples from the second batch of 11 animals was extracted and prepared by RNeasy mini Kit (Qiagen), according to the manufacturer's protocol. Reverse transcription was performed by using the Superscript II First Strand DNA synthesis system (Invitrogen). Quantitative PCR was performed using SYBR Green I Master Mix Buffer (Applied Biosystems, Foster City, CA, USA), and reactions were run on a SDS7000 (Applied Biosystems, Foster City, CA, USA) using a three-step standard protocol. β-actin was used as an internal standard, and ΔCT values were calculated from differences between the analyzed genes and β-actin. The following primer pairs were used: CTGCTGGACTTCTCTACGGAT (forward), CAGTAGAGGTGGAAGGATGGG (reverse).

### Statistics

Data were analyzed using one-way ANOVAs with prenatal treatment as a between-subjects factor (sham vs. MAM), except for the number of heterotopias and discontinuities that were analyzed using the Mann-Whitney U test of rank. Methylation levels were compared by a two-way ANOVA analysis (CpG site and group as factors). For global methylation and reelin expression levels, the two groups were compared using the Mann-Whitney non parametric test. Level for significance was set at p<0.05.

## Results

The methodology followed was mostly determined by the fact that considerable amounts of data coming from postmortem brains of patients with schizophrenia are performed via two-dimensional (2D) techniques (Tables 1 and 2). In addition, both methods, two-dimensional as well as three-dimensional carry their own flaws [54] and if these confounders are not taken into account significant biases emerge [55], [56]. As will be discussed in the later sections of this manuscript, particular attention was paid to avoid as much as possible potential artifacts that could mislead our results.

**Table 1:** Neuronal size and density in the hippocampal formation of MAM-treated rats and patients with schizophrenia.

Anatomical area in MAM^1^-treated rats	Findings	Comparable studies in schizophrenia	Anatomical Area	Findings
Hippocampal subfields 2CA1 and CA4	Unchanged neuronal density by 2D^3^ methods	Falkai and Bogerts, 1986	CA1 and CA4	Decreased neuronal density by 2D methods
		Jeste and Lohr, 1989	CA1 and CA4	Decreased neuronal density by 2D methods
		Heckers et al, 1991	CA1 and CA4	Unchanged neuronal density by 3D^4^ methods
		Benes et al, 1991	CA1	Decreased neuronal density by 2D methods
		Arnold et al, 1995	CA1 and CA4	Unchanged neuronal density by 2D methods
		Zaidel et al, 1997a	CA1 (right)	Increased neuronal density by 2D methods
		Benes et al, 1991	CA4	Unchanged neuronal density by 2D methods
		Zaidel et al, 1997a	CA4	Unchanged neuronal density by 2D methods
		Walker et al, 2002	CA1 and CA4	Unchanged neuronal density by 3D methods
		Schmitt et al, 2009	CA1 and CA4	Unchanged neuronal density by 3D methods
Hippocampal subfield CA2	Decreased neuronal density by 2D methods	Falkai and Bogerts, 1986	CA2	Decreased neuronal density by 2D methods
		Jeste and Lohr, 1989	CA2	Decreased neuronal density by 2D methods
		Heckers et al, 1991	CA2	Unchanged neuronal density by 3D methods
		Benes et al, 1991	CA2	Unchanged neuronal density by 2D methods
		Arnold et al, 1995	CA2	Unchanged neuronal density by 2D methods
		Walker et al, 2002	CA2	Unchanged neuronal density by 3D methods
		Schmitt et al, 2009	CA2	Unchanged neuronal density by 3D methods
Hippocampal subfield CA3	Increased neuronal density by 2D methods	Falkai and Bogerts, 1986	CA3	Decreased neuronal density by 2D methods
		Jeste and Lohr, 1989	CA3	Decreased neuronal density by 2D methods
		Heckers et al, 1991	CA3	Unchanged neuronal density by 3D methods
		Benes et al, 1991	CA3	Unchanged neuronal density by 2D methods
		Arnold et al, 1995	CA3	Unchanged neuronal density by 2D methods
		Zaidel et al, 1997a	CA3 (right)	Increased neuronal density by 2D methods
		Walker et al, 2002	CA3	Unchanged neuronal density by 3D methods
		Schmitt et al, 2009	CA3	Unchanged neuronal density by 3D methods
Hippocampal subfields CA3 and CA4	Decreased neuronal soma size by 2D methods	Benes et al, 1991	All CA subfields	Decreased neuronal soma size by 2D methods
		Arnold et al, 1995	All CA subfields	Decreased neuronal soma size (but only significant for CA1) by 2D methods
		Zaidel et al, 1997b	All CA subfields	Decreased neuronal soma size by 2D methods
		Jonsson et al, 1999	All CA subfields	Decreased neuronal soma size by 2D methods
		Highley et al, 2003	All CA subfields	Unchanged neuronal soma size by 3D methods

1 Methylazoxymethanol during embryonic day 17.

2 Cornu ammonis.

3 Two dimensional.

4 Three dimensional.

**Table 2:** Neuronal size and density in the entorhinal cortex of MAM-treated rats and patients with schizophrenia.

Anatomical area in MAM^1^-treated rats	Findings	Comparable studies in schizophrenia	Anatomical Area	Findings
ERC^2^	Unchanged neuronal density by 2D^3^ methods	Falaki et al, 1988	ERC	Decreased neuronal density by 2D methods
		Arnold et al, 1995	ERC	Unchanged neuronal density by 2D methods
		Krimer et al, 1997	ERC	Unchanged neuronal density by 2D methods
ERC	Decreased neuronal soma size by 2D methods	Jakob and Beckmann, 1994	ERC	Decreased neuronal soma size (qualitative)*
		Arnold et al, 1995	ERC	Decreased neuronal soma size by 2D methods

1 Methylazoxymethanol during embryonic day 17.

2 Entorhinal cortex.

3 Two dimensional.

*All other studies were quantitative.

### Morphometric effects of MAM E17 treatment on brain structures

**Figure 1:**
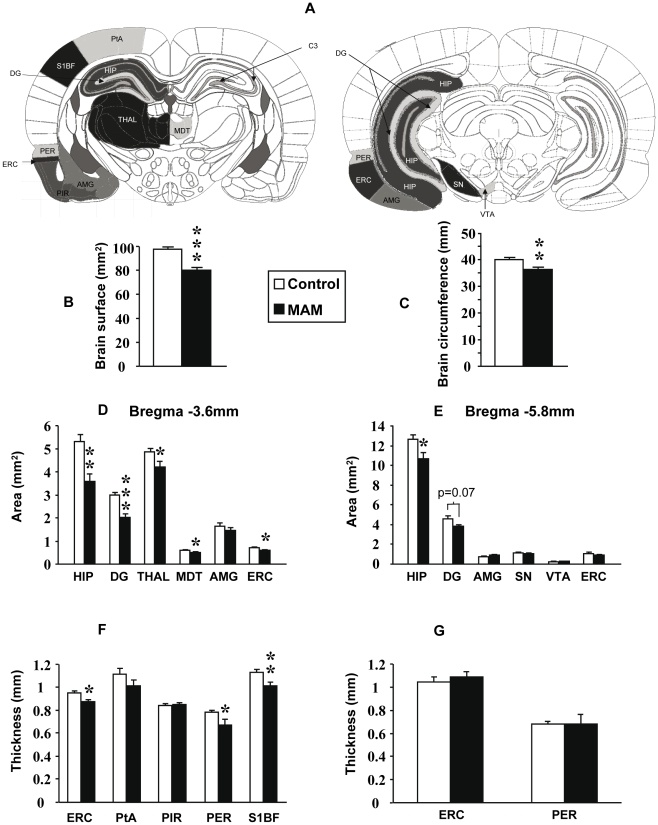
Morphometric effects of MAM E17 treatment on brain structures. (A) Brain diagrams reproduced with permission from The Rat Brain Atlas by Paxinos and Watson 6 edition illustrating how anatomical areas were delineated. Bregma −3.8mm on left and Bregma −5.8mm on right. Areas and thicknesses were measured as delineated in the pictures. (B–C) Graphs indicating differences in brain area (B) and circumference (C) between control (white bars) and MAM-exposed animals (black bars). (D–E) Graphs indicating area differences between MAM and control animals at Bregma −3.6 (D) and −5.8mm (E). (F–G) Graphs indicating differences in thickness between MAM and control animals at Bregma −3.6 (F) and −5.8mm (G). Results represent the mean ± S.E.M. of 9 animals per group from which slides done by duplicate were analyzed. Amygdala (AMG), associative parietal cortex (PtA), cornu ammonis (CA), dentate gyrus (DG), entorhinal cortex (ERC), hippocampus (HIP), mediodorsal thalamic nucleus (MDT), perirhinal cortex (PER), piriform cortex (PIR), somato-sensory cortex barrel field 1 (S1BF), substantia nigra (SN), thalamus (THAL), ventral tegmental area (VTA). *p<0.05 **p<0.01 and ***p<0.001.

The general appearance of MAM-exposed brains did not differ from that of control animals, although MAM-exposed brains appeared slightly smaller by simple observation. This characteristic was confirmed after measuring brain area and circumference at Bregma −3.6mm (Figure 1A) as both analyses rendered statistically significant diminutions (Figure 1B and C) (p<0.001 and p<0.01 respectively). In addition, there was a reduction in the cortical volume that was more pronounced in the posterior cortex where the subcortical colliculi becomes exposed.

Subsequently, we sought morphometric changes at Bregma −3.6mm and Bregma −5.8mm levels through combined Nissl and luxol staining (Kluver-Barrera technique), which highlights myelin, fibre bundles and cell nuclei. Area and thickness of the anatomical structures studied were measured as shown on Figure 1A. At Bregma −3.6mm, MAM-exposed brain areas were significantly reduced in the HIP (p<0.01), DG (p<0.001), THAL (p<0.05), MDT (p<0.05) and ERC (p<0.05) (Figure 1D). Likewise, the thickness of the ERC, PER and S1BF cortices was significantly decreased (p<0.05, p<0.05 and p<0.01 respectively) when compared to controls (Figure 1F). In contrast, neither the AMG, PtA nor PIR cortex showed any differences in area or in thickness at this level (Figures 1D and F). Bregma −5.8mm level analysis also revealed a significant decrease in the hippocampal area (p<0.05) and a tendency towards a significantly smaller DG (p = 0.07) (Figure 1E). The other structures studied, including the AMG, ERC and VTA did not show any changes at Bregma −5.8mm (Figure 1E). ERC and PER thickness at the same level also did not reveal any differences (Figure 1G).

### Cytoarchitectural and neuromorphometric alterations after MAM E17 treatment

The neuronal density and neuronal soma size were examined in all the anatomical structures revealing decreased area or thickness in MAM-treated rats. However, the neuronal cytoarchitecture was only studied in the hippocampal formation (ERC plus all the hippocampal subfields) because it is in this brain region where cytoarchitectural anomalies have been reported in schizophrenia [57] and because this is the targeted brain region at E17.

**Figure 2:**
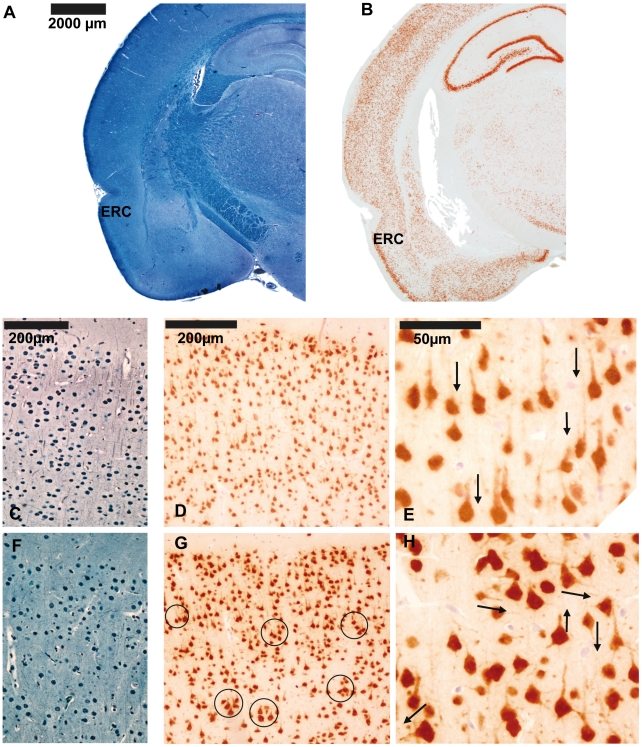
Neuronal organization of entorhinal cortex in MAM and control rats. Coronal sections of the entorhinal cortex at Bregma −3.8mm from control animals stained with Bodian-Luxol (A) and NeuN immunostaining (B). (C) Bodian-Luxol staining of the entorhinal cortex from control animals. A well defined columnar organization and myelin with parallel directions can be observed. Arrows point to myelin pathways. (D) Anti-NeuN immunostaining of the entorhinal cortex from control animals showing axon hillocks with a parallel orientation and neuronal bodies regularly organized. (E) Neuronal somas and axon hillocks are clearly distinguished in this picture magnified 400×. Arrows point to axon-hillock direction. (F) Bodian-Luxol staining of the entorhinal cortex from MAM rats reveals a less obvious columnar organization while myelin direction appears random. (G) NeuN immunostaining of the entorhinal cortex from MAM animals. Neuronal disorganization with clusters of neurons is evident mainly in layers II and III. Circles highlight neuronal clusters. (H) Neuronal disorganization, neuronal size variability and axon hillocks with random directions can be observed in this picture magnified 400×. Arrows point to axon-hillock direction.

In order to assess neuronal organization, density, size and morphology in the entorhinal cortex, two staining methods were used (Figures 2A and B). One method was Bodian-Luxol, which highlights myelin and neuronal soma (Figure 2A). The other marker utilized was through anti-NeuN antibodies that bind specifically to the DNA-binding protein NeuN (Figure 2B). This protein is specific for neurons and is restricted to the axon hillock, the neuronal soma and the nucleus. These staining methods evidenced well defined layers in the entorhinal cortex of control animals where somas had a columnar organization and axon hillocks had parallel directions (Figures 2 C, D and E). In contrast, in MAM-exposed brains, cellular organization in this same anatomical area was less strict and axons with perpendicular orientation could be seen (Figures 2 F, G and H). In addition, columns were less obvious and neuronal clusters were present. In some animals, large isolated neurons could be identified (Figures 2 C, D and E). Neuronal clusters were mostly found in layers II and III of the ERC of MAM-treated rats (Figures 2 C, D and E).

**Figure 3:**
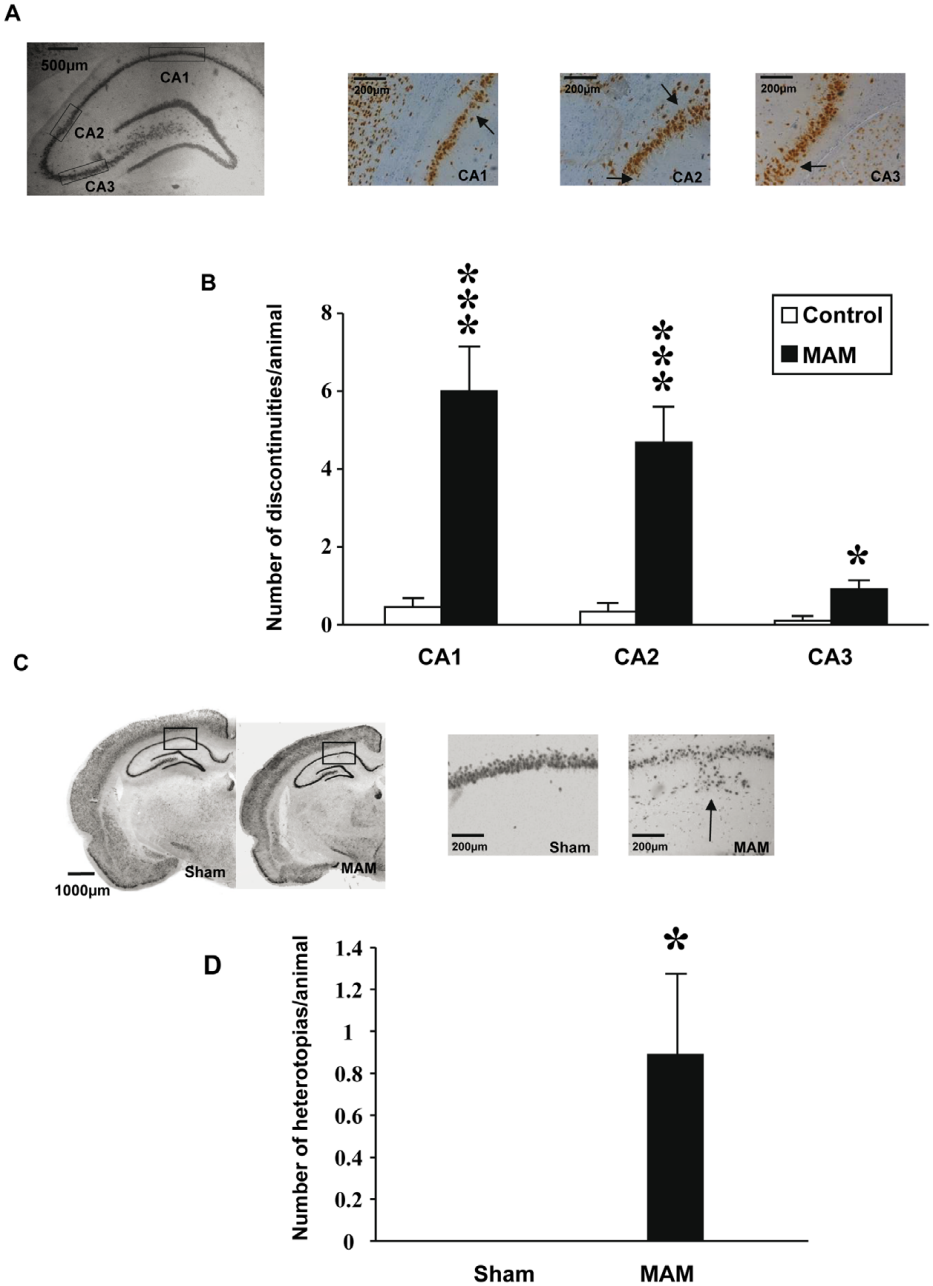
Discontinuities and heterotopias in MAM and control animals. (A) Coronal slices at Bregma −3.8mm of the hippocampus, CA1, CA2 and CA3 from MAM exposed rats stained with anti-NeuN antibody and 3,3′-diaminobenzidine as chromogen. The rectangles in the first photo indicate where the magnification shown in the three following photos comes from. Arrows point to actual discontinuities. (B) Graph bars indicating discontinuity differences between MAM rats and control animals in hippocampal subfields CA1, CA2 and CA3. (C) Coronal slices at Bregma −3.8mm from MAM and control animals stained with anti-NeuN antibody and 3,3′-diaminobenzidine as chromogen. The rectangles within the two left photos indicate where the magnification shown in the two right photos comes from. The arrow points to an actual heterotopia. (D) Graph indicating number of heterotopias in MAM rats' hippocampus compared to control animals. Results represent the mean ± S.E.M. of 9 animals per group from which slides done by duplicate were analyzed. *p<0.05 and ***p<0.001.

**Figure 4:**
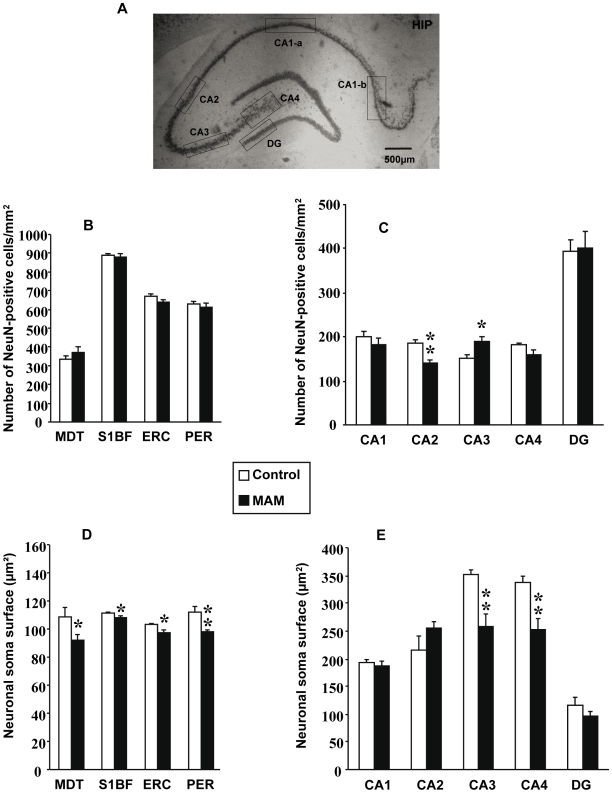
Neuronal density and neuronal soma size in MAM and control animals. (A) Coronal section at Bregma −3.8mm stained with anti-NeuN antibody and 3,3′-diaminobenzidine as chromogen indicating how hippocampal subfields were delineated. (B) Graph indicating changes in neuronal densities from MDT, VPM, VPL, S1BF, PER and ERC between MAM (black bars) and control animals (white bars). (C) Graph indicating changes in neuronal densities between MAM and control animals from hippocampal subfields CA1, CA2, CA3, CA4 and DG. (D) Graph indicating changes in neuronal soma size from MDT, VPM, VPL, S1BF, PER and ERC between MAM and control animals. (E) Graph indicating differences between MAM and control animals' cross-sectional area of neuronal somas from CA1, CA2, CA3, CA4 and DG. Results represent the mean ± S.E.M. of 9 animals per group from which slides done by duplicate were analyzed. In graphs D and E individual values are the mean of at least 500 measured neurons for each cerebral structure or hippocampal layer *p<0.05 and **p<0.01.

Discontinuities and heterotopias in the dorsal hippocampus were also quantified using NeuN immunostaining (Figure 3). MAM-exposed rats exhibited more discontinuities in CA1 (p<0.001), CA2 (p<0.001), and CA3 (p<0.05) (Figures 3A and B). The number of heterotopias present in the dorsal hippocampus was also more common in MAM rats (p<0.05) (Figures 3C and D). Moreover, the neuronal density was significantly decreased in CA2 (p<0.01) whereas the opposite was observed in CA3 (p<0.05) of exposed animals (Figures 4A and C). No differences in the neuronal density were observed in subfields CA1, CA4 or DG (Figure 4C). Likewise, no differences in neuronal soma size were found in CA1, CA2 or DG whereas significant reductions were encountered in CA3 and CA4 (both p<0.01) (Figure 4E).

The neuronal density did not differ between MAM and control rats in the MDT, S1BF, ERC or PER (Figure 4B). On the other hand, the neuronal soma size was significantly decreased in the MDT, S1BF, ERC and PER (all p<0.05 except PER p<0.01) (Figure 4D).

### Ventricular enlargement induced by MAM E17 treatment

**Figure 5:**
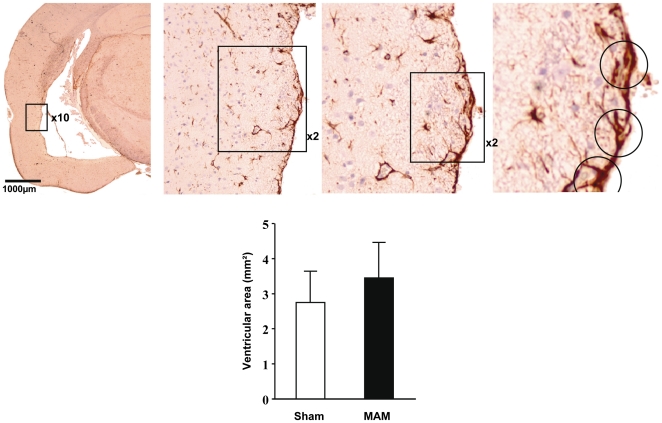
Ventricular size in MAM and control animals. (Top left) Coronal section from a representative MAM rat at Bregma −3.8mm stained with anti-GFAP antibodies and 3,3′-diaminobenzidine used as chromogen. An enlarged ventricle could be observed in the picture. (Top center and top right) Three magnifications (10×, 2× and 2×) are shown to evidence tanycytes, which are circled in the last magnification. (Bottom) Graph indicating differences in ventricular size between MAM (black bars) and control animals (white bars). Results represent the mean ± S.E.M. of 9 animals per group from which slides done by duplicate were analyzed.

An obvious enlargement of the lateral ventricles could be seen in several MAM rats. Nonetheless, this effect was not statistically significant likely due to its not systematic presence (p>0.05) (Figure 5). To verify that the ventricle enlargement was not due to a freezing artefact, the integrity of the ventricular epithelium was analyzed. Anti-GFAP immuno-staining was performed in order to reveal tanycytes, which are specialized astrocytes localized at the border of ventricles (Figure 5). The presence of these cells is consistent with an intact ventricular epithelium. The subpial layer is easily distinguishable, vascular and lepto-meningeal endings of astrocytes highlight the presence of blood vessels and delineate brain tissue borders. Tanycytes were well differentiated from protoplasmic astrocytes through their stellar arborizations. In MAM rats, the presence of tanycytes bordering the ventricles confirmed that the ventricular enlargement observed was effectively due to atrophy of underlying structures and not to a freezing artefact. Therefore, ventricular enlargement was a consequence of MAM exposure but as mentioned above its inconsistent presence rendered it non-significant (Figure 5).

### Hippocampal quantification of reelin expression, methylation and reelin containing cells

**Figure 6:**
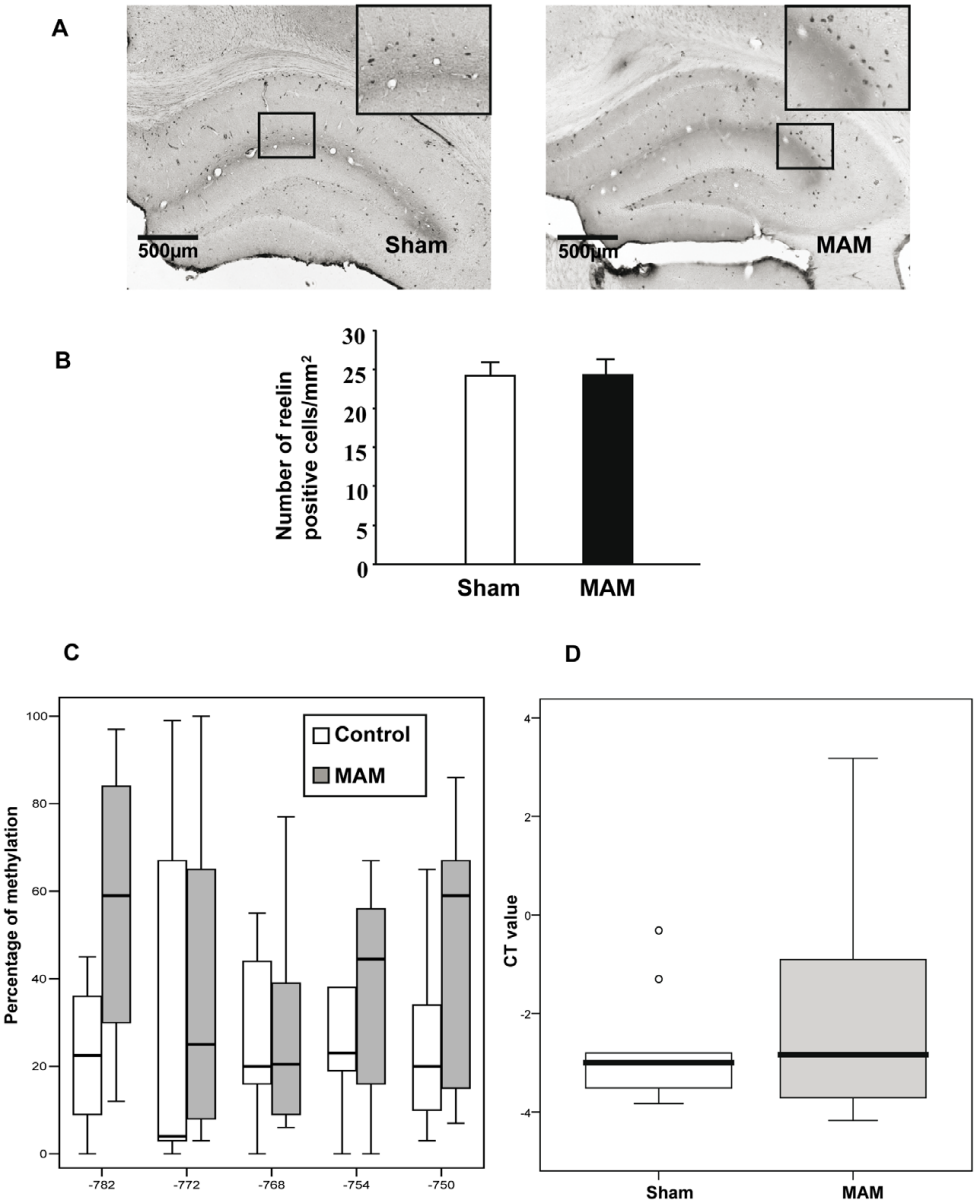
Reelin-containing cells, reelin expression and methylation in the hippocampus of MAM and control animals. (A) Coronal slices from MAM and control animals at Bregma −3.8mm stained with anti-reelin antibodies and 3,3′-diaminobenzidine used as chromogen. (B) Graph indicating differences in reelin positive cells between MAM (black bar) and control animals (white bar). Results represent the mean ± S.E.M. of 9 animals per group from which slides done by duplicate were analyzed. (C) Graph bars indicating methylation differences between MAM and control animals in five CpGs (−782, −772, −768, −754, and −750) from the reelin promoter within the hippocampus. Methylation levels are expressed in percentage for Sham and MAM groups by each measured CpG site. (D) Graph bars indicating differences in reelin mRNA expression from MAM and control rats' hippocampus. Reelin mRNA expression levels were plotted as delta CT values.

Reelin containing cells were assessed in the hippocampus at Bregma −3.8mm level (Figure 6A). There were no differences in the number of reelin positive cells between MAM and control animals (Figure 6B). Since the number of reelin-containing cells does not exclude a potential deficit in reelin's expression or methylation a different batch of MAM and control animals was used to pursue these assessments.

The amplification of a 252 bp sequence located in the reelin promoter allowed the determination of the methylation level of five CpGs situated at −782, −772, −768, −754, and −750 from the first base of exon 1. ANOVA analysis embracing the CpG site and group as factors revealed only a tendency towards hypermethylation of the reelin promoter in MAM-treated rats (F (1,74) = 3.2, p = 0.075) (Figure 6C). However, global methylation levels computed through means of the median of the five CpG sites for each animal did not vary between the two groups (U-Mann Whitney = 9, p = 0.34). Moreover, reelin expression levels did not change between MAM and control animals (U-Mann Whitney = 25, p = 0.81) (Figure 6D).

## Discussion

### Methodological Considerations

Within the neuroscientific community it is commonly believed that three-dimensional (3D) assessments provide more accurate results than 2D methods. However, both methodologies have their own flaws [54] and when these artifacts are not considered stereological 3D analyses could involve biases of up to 80% [55] while the consideration of potential confounders leaves 2D methods 2% short of brain section reconstructions [56]. One confounder concerning stereological 3D methods involves brain section shrinking that affects the edges more than the center of the slice [54]. This non-linear distortion can directly alter the neuronal density as well as the neuronal soma size [54], both of which represent the focus of our analyses.

Two-dimensional techniques are by no means error-free. In fact, the main confounder associated with the establishment of cell numbers via 2D methodologies is cell over-counting. Biases are introduced by counting soma cells more than once. This potential artifact could be partially corrected by using the Abercrombie equation [58] but irregularities in cell size and shape, as in the case of the MAM model, limit the utility of such tool [59]. We did not count any consecutive brain slices to avoid over-counting but we did count at least 3 sections per level to reduce biases introduced by potential changes in the shape of brain regions. As can be seen in Figures 4B and C, the technique we followed gave quite consistent results for neuronal numbers. The fact that we found a decreased as well as an increased cell density in certain brain regions of MAM-treated animals also indicates that there was not an obvious over-counting of cells in either group. Moreover, these density changes are in agreement with MAM effects as will be discussed in the following sections. On the other hand, when the neuronal soma size is under investigation, 2D methods have to rely on finding the neuronal nucleolus to measure the main cell circumference and avoid measuring an unevenly cut neuronal body. The number of cells analyzed can also limit such a confounder. It has been shown that lager sample sizes decrease the likelihood of a biased result [60]. Consequently, we counted between 400–500 cells per condition. Moreover, our results concerning the neuronal soma size are again in line with MAM properties as will be explained below. Additionally, a 2D approach was used because the main goal of this work is to determine if hippocampal formation size reductions found in schizophrenia and the MAM model are caused by similar neuropathological findings and most of the studies concerning this brain region in affected individuals have been done via 2D methods (Tables 1 and 2).

### Neuropathological findings in MAM E17 rats and schizophrenia; differences and similarities

Several features found in E17 MAM-exposed rat brains resemble those described in the brains of patients with schizophrenia, in particular the overall decrease in cortical size. In schizophrenia, however, this cortical reduction is only evident after meticulous analyses [61], [62] whereas in MAM-treated animals it is noticeable even through gross brain inspections [63]. More specific examinations revealed size reductions in a restricted number of brain regions as well as an obvious enlargement of the lateral ventricles in several MAM rats but ventricle enlargement, one of the most consistent findings in schizophrenia [64], [65], was not statistically significant in MAM-exposed animals (Figure 5).

The subcortical area most commonly associated with schizophrenia is the thalamus, in particular the MDT. Both structures have consistently shown volumetric reductions in affected individuals [66]–[70] albeit not always [71]–[73]. Likewise, the MAM model evidences a smaller thalamus and has now replicated data on MDT size reductions [45] (Figure 1D). Neuromorphometric analyses indicate that the neuronal soma diminutions reported by some authors in the MDT from patients with schizophrenia [68] are also found in MAM-treated animals (Figure 4A) (Table 3). When it comes to neuronal numbers in this brain region, the literature is divided (Table 3). Five studies have reported a decreased neuronal density in the MDT of patients with schizophrenia [67], [68], [74]–[76] whereas four recent reports, arguably following stricter methodologies, showed an unchanged neuronal density in the MDT [66], [71]–[73] (Table 3). In MAM-exposed brains we found no changes in the neuronal density in this thalamic nucleus (Figure 4B) but no hard conclusions can be drawn regarding potential differences or similarities with schizophrenia owing to discrepancies in the literature concerning individuals with this psychotic illness (Table 3).

**Table 3:** Neuronal size and density in the mediodorsal thalamus of MAM-treated rats and patients with schizophrenia.

Anatomical area in MAM^1^-treated rats	Findings	Comparable studies in schizophrenia	Anatomical area	Findings
MDT^2^	Unchanged neuronal density by 2D^3^ methods	Pakkenberg, 1990	MDT	Decreased neuronal density by 3D^4^ methods
		Popken et al, 2000	MDT	Decreased neuronal density by 3D methods
		Young et al, 2000	MDT	Decreased neuronal density by 3D methods
		Thune and Pakkenberg, 2000	MDT	Decreased neuronal density by 3D methods
		Byne et al, 2002	MDT	Decreased neuronal density by 3D methods
		Cullen et al, 2003	MDT	Unchanged neuronal density by 3D methods
		Dorph-Petersen et al, 2004	MDT	Unchanged neuronal density by 3D methods
		Danos et al, 2005	MDT	Unchanged neuronal density by 3D methods
		Kreczmanski et al, 2007	MDT	Unchanged neuronal density by 3D methods
MDT	Decreased neuronal soma size by 2D methods	Byne et al, 2002	MDT	Decreased neuronal soma size by 3D methods
		Cullen et al, 2003	MDT	Unchanged neuronal soma size by 3D methods
		Dorph-Petersen et al, 2004	MDT	Unchanged neuronal soma size by 3D methods

1 Methylazoxymethanol during embryonic day 17.

2 Mediodorsal thalamus.

3 Two dimensional.

4 Three dimensional.

The somatosensory cortex from MAM rats also evidences smaller neuronal somas (Figure 4D), suggesting that dendrites and axons are shorter [77]–[79] and consequently connectivity is expected to be impoverished. These results are in line with functional neuroimaging studies indicating that somatosensory cortex activation is reduced in patients with schizophrenia [80], [81]. Nonetheless, to our knowledge there are no neuroanatomical reports from this cortical zone in schizophrenia and thus our results in MAM rats will have to await correlation. The size of the amygdala on the other hand, remains unchanged in MAM-exposed animals (Figures 1D and E), which correlates with recent postmortem and neuroimaging data from patients with schizophrenia [17], [82]–[84] although opposite results have also been published [85].

Within the cortex, the largest body of evidence associates the hippocampal formation, along with the dorsolateral-PFC, with schizophrenia [41]. Neuropathological studies have contributed considerably to this association. For instance, the dentate gyrus from patients with this psychotic disorder has shown higher numbers of granular cells with basal dendrites [86], [87] but potential deficiencies in its entire volume and its cytoarchitecture are still to be determined. Therefore, our findings involving a size reduction in the dentate gyrus (Figures 1D and E) do not strictly parallel what is currently known in schizophrenia. More detailed investigations embracing dendrite morphology in MAM-treated rats are warranted. In contrast, the hippocampus and the perirhinal cortex from MAM rats are reduced in size (Figures 1D, E and F), which corroborates previous findings from MAM exposure at E17 [43], [45] as well as earlier in embryonic life (E9–E12) [30], [31]. These results parallel the hippocampal and perirhinal cortex volume loss consistently found in schizophrenia [19], [64], [85], [88]–[92] but in themselves these results are not specific. Brain volume reductions could originate from a variety of causes and only cytoarchitectural and neuromorphometric analyses could establish if the MAM model does indeed replicate hippocampal anomalies found in schizophrenia (Table 1). A more detailed assessment revealed that the neuronal density is decreased in CA2, increased in CA3 and unchanged in CA1 and CA4. Similar results have been found in schizophrenia where CA2 density was decreased [93], [94], CA3 increased [95] and CA1 and CA4 unchanged [13], [96]–[98]; however, other reports suggest that there are no changes in CA2 and CA3 cell density [12], [13], [96]–[98] and even that CA3 density is decreased [94]. Likewise, data on CA1 is inconsistent, with some studies pointing to unchanged [12], [96]–[98], increased [95] and decreased density [13], [94]. Thus, the reliability of schizophrenia findings concerning hippocampal cell density is limited making any comparisons dubious. More consistent findings come from neuronal organization although controversy surrounding these results persists [41]. Neuronal disorientation has been reported in several studies [99]–[102], albeit negative reports have also been published [12]–[14], [103]. In the MAM model, gross inspection reveals a high degree of disorientation in hippocampal neurons. But perhaps the most consistent finding in the hippocampus of patients with schizophrenia concerns the neuronal soma size (Table 1). Only one team has not found changes in the hippocampal neuronal soma size [104], whereas three independent groups have reported neuronal soma size reductions in all the CA subfields of affected individuals [12]–[15]. MAM-treated rats replicate such findings in hippocampal subfields CA3 and CA4 (Figure 4E).

The entorhinal cortex in psychotic individuals has shown whole size reductions [11], [18], [19] along with laminar disorganization, reduced neuronal soma size and the presence of neuronal clusters [3], [4], [12], [16]. All such findings are reproduced by the MAM E17 model to different extents (Table 2). Aberrantly clustered neurons can be seen in layers II and III of MAM-treated animals (Figure 2G). Layers II and III are also the most affected in schizophrenia when it comes to abnormal neuronal clusters [3]–[6]. Layer disorganization in the ERC is also notably affected in both entities [3], [4] (Figure 2F). Additionally, heterotopias are present in the entorhinal cortex of patients with schizophrenia [3], [4], [5], whereas abnormally located neurons in the MAM model were only seen in the hippocampus (Figures 3A, B, C and D). However, all these findings could result from intrinsic anatomical variations in the ERC [57]. In contrast, clear deficiencies were found by assessing the neuronal density and the neuronal soma size (Table 2). Neuronal density in the ERCs of affected individuals has either been found to be decreased [18] or unchanged [9], [12]. We found that neuronal density was unchanged in the ERC of MAM-exposed rats (Figure 4B). The size of this brain region is reduced in schizophrenia [11], [17]–[19] and the neuronal soma is diminished [12], [16]. Likewise, in MAM-treated rats, entorhinal cortex area and thickness were decreased (Figures 1D and F) and this size reduction was associated with a significant neuronal soma shrinkage (Figure 4D). Other teams have shown that MAM exposure earlier in embryonic life (E9–E12) also causes ERC size reductions at different bregma levels and that also compromises the entorhinal cytoarchitecture [30]–[32]. Thus, a number of neuropathological similarities within the hippocampal formation can be found between MAM-exposed rats and patients with schizophrenia. The possible physiological implications of these neuropathological coincidences will be discussed in the following sections.

### MAM mechanisms of action and its implications for cytoarchitecture, neuronal soma size and neuronal density

MAM is mostly known as an antimitotic agent [36]–[38] while its capacity to affect neurite outgrowth is less recognized [105], [106]. However, it has been previously shown that dissociated hippocampal neurons from embryonic day 18 exposed to MAM are unable to form axons or dendrites and that the few axons already present retract [105]. Moreover, MAM significantly reduces microtubule associated proteins (MAPs) 2 and 1B [105], two proteins essential for neurite sprouting [107]. These results are further supported by experiments *in vivo* in which the hippocampus of animals treated with MAM at embryonic day 15 evidenced less and shorter dendritic branches, abnormal dendritic shafts and diminished spine numbers [106]. Our results, after MAM exposure at embryonic day 17, appear to involve a combination of both effects; that of an antimitotic agent and an inhibitor of neurite formation. This is not surprising since at embryonic day 17 neurogenesis peaks in the hippocampal formation [39] and it is also during this day when fibers interconnecting this brain region are extended [40].

A closer look at each of the anatomical areas affected reveals which mechanism was most influential. In the hippocampal subfield CA2 an antimitotic effect appears to predominate since the number of neurons decreased and there was no change in the neuronal soma size (Figures 4C and E). All the other anatomical regions affected exhibited deficits consistent with arrested neurite formation as they presented decreased neuronal soma size and as previously mentioned, neuronal soma size correlates with neuropil length [77]–[79]. These anatomical regions include S1BF, MDT, ERC, PER CA3 and CA4 (Figures 4D and E). CA3 is a particular case because its neuronal density was also increased (Figure 4C). A higher neuronal density coupled with a reduction in the anatomical area size of, in this case, the hippocampus (Figures 1E and F) indicates that the space between neurons is reduced [108]. Since this space is usually occupied by dendrites and axons, its reduction suggests that the neuropil is shrunken [108]. Thus, CA3's impoverished neuropil is supported by two lines of information; neuronal density and neuronal soma size (Figures 4C and E).

How MAM exerts its antimitotic and neurite outgrowth inhibitory effects is still to be determined; one possibility is through DNA methylation, a mechanism known to regulate gene expression. MAM's capacity to induce gene-methylation [36] could affect reelin. Partial knockouts have shown that under-expressing reelin during rodents' development leads to neuronal migration deficits [51] and to shorter hippocampal dendrites in adulthood [49]. The phenotypic coincidences between partial knockouts of the reelin gene and MAM-exposed animals suggest reelin might be implicated in MAM effects. In addition, reelin has been consistently associated with schizophrenia where its expression and immunoreactivity were found to be decreased [23]–[25]. Besides, hypermethylation of the reelin promoter in patients with schizophrenia has been reported [26], [27]. Nonetheless, the most recent study concerning reelin methylation in schizophrenia found no differences with control individuals [29].

Our results indicate that reelin is not significantly affected in MAM-treated rats. However, it has to be kept in mind that our sample is rather small and that we measured reelin methylation and expression in the entire hippocampus (Figures 6C and D). Since neuropathological deficits were found in specific hippocampal regions and not in the entire hippocampus a similar by region analysis of reelin methylation and expression levels is warranted before excluding reelin as a molecule involved in MAM's mechanism of action. It also has to be considered that MAM could exert its effects by methylating other genes. A number of genes involved in neurite outgrowth with replicated evidence of their association with schizophrenia [109] could be potentially involved in MAM's neuropathological consequences. Further research following these leads is being developed by our group as we believe significant insight can be gained into the pathophysiology of schizophrenia if abnormal neurite formation, a proposed etiological factor for this illness [109], is factored into the equation.

### Plausible implications and future directions of MAM neuropathological findings and its relation to schizophrenia

The MAM model presents reductions in neuronal soma size within the entorhinal cortex, CA3 and CA4 that resemble post-mortem findings from schizophrenic patients (Table 1). These soma size deficiencies suggest the dendritic arbor and the axonal length are impoverished [77]–[79]. Consequently, efferents leaving these anatomical structures are likely to be compromised. The entorhinal cortex sends connections to the hippocampal formation [110] and to limbic structures [111] including the amygdala [112] and the nucleus accumbens [113] while the hippocampal subfields CA3 and CA4 only extend fibers within the hippocampal formation [53]. Given that connections between the entorhinal cortex and the hippocampus are an important memory center, it is likely that the deficits encountered in the hippocampal formation contribute to MAM rats' cognitive deficits [35], [42], [43]. This could be particularly true for visuo-spatial anomalies as it was recently suggested that the entorhinal cortex is the substrate for visuo-spatial representations [114]. Thus, it is not surprising that both entities, schizophrenia and MAM-exposed animals, present neuropathological anomalies in the entorhinal cortex (Table 2) as well as visuo-spatial deficiencies [115]–[117]. The potential association between ERC anomalies and visuo-spatial deficits is a research line to be followed up in MAM-treated animals.

Another plausible association between our findings in MAM-exposed animals and patients with schizophrenia comes from the potential decline of efferents leaving the entorhinal cortex to reach the hippocampal subfields including the dentate gyrus [110]. These fibers are essentially glutamatergic [118] and thus their shrinkage in the E17 MAM model, suggested by a reduction in the neuronal soma size (Figure 4D), would be in line with the hypoglutamatergic hypothesis of schizophrenia. Nonetheless, evidence of direct glutamatergic deficits in the MAM E17 model is still lacking.

Also relevant to schizophrenia is the infraconnectivity between the entorhinal cortex and limbic structures [111]–[113] presumably present in MAM rats (Figure 5A). In humans, impaired limbic connectivity is thought to be implicated in the negative symptoms observed in schizophrenia. MAM-treated rats show behaviors that parallel negative symptoms such as deficits in social interaction [42], [43]. Thus the MAM model provides the opportunity to further investigate a potential association between deficient ERC projections to limbic structures and social interaction anomalies.

In conclusion, we can say that even though not all the neuropathological findings encountered in patients with schizophrenia are replicated by the MAM E17 model and vice versa, the findings presented here indicate that there are significant similarities in the hippocampal formation of both conditions. Of note, these deficits point to abnormal neurite formation, a recently proposed etiological factor for schizophrenia [109]. Finally, our results along with their implications in cognition and behavior provide further support for the MAM E17 model as a useful tool for studying the pathophysiology of this psychotic disorder.
